# Engineering Ferrate(VI) for Water and Wastewater Treatment: Translating Science into Technology

**DOI:** 10.1021/acs.est.5c16821

**Published:** 2026-04-14

**Authors:** Zepei Tang, Zhenli He, Yang Deng

**Affiliations:** † Department of Earth and Environmental Studies, 8087 Montclair State University, Montclair, New Jersey 07043, United States; ‡ Department of Soil, Water and Ecosystem Sciences, Indian River Research and Education Center, University of Florida-IFAS, Fort Pierce, Florida 34945, United States

**Keywords:** valley of death, technology development, ferrate(VI) generation, ferrate(VI) activation, process intensification, chemical oxidants, water treatment, wastewater treatment

## Abstract

Over the past five decades, ferrate­(VI) (FeO_4_^2–^) for water and wastewater treatment has remained largely at the laboratory scale, with limited pilot- and full-scale implementation. This gap between sustained research and scarce real-world deployment places the technology in the Valley of Death—a high-risk stage of development—and highlights the need for an engineering-centric approach to enable practical application. This article presents an engineering framework to guide the development and translation of ferrate­(VI) technology for water and wastewater treatment. From a system perspective, ferrate­(VI) technology comprises three core subsystems: generation, treatment, and solid–liquid separation. Technology maturity depends on both the advancement of individual subsystems and their effective integration. Key obstacles and corresponding engineering strategies are identified for each subsystem. Among them, ferrate­(VI) generation, despite commercial availability, remains the primary bottleneck due to limited industrial adoption driven by challenges related to chemical instability, residual impacts, and energy demand. As subsystem maturity progresses, system-level integration emerges as a critical challenge, requiring alignment among subsystems or with existing infrastructure under different implementation configurations. To advance ferrate­(VI) technology across the Valley of Death, we emphasize maturity stage–aligned development, integrated system design, and application-specific decision frameworks.

## Graphical abstract

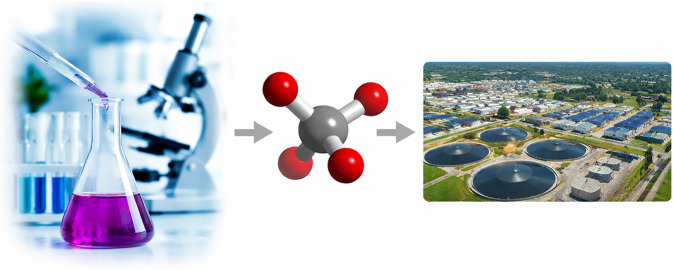

## 1 Ferrate(VI) Technology: Maturity, Barriers, and the Need for Engineering

Ferrate­(VI) (FeO_4_^2–^), an Fe­(VI) oxoanion,^1,2^ has been investigated for water and wastewater treatment since the 1970s.^3–5^ Its strong oxidative reactivity, multifunctionality, and minimal formation of disinfection byproducts (DBPs) offer potential for diverse treatment applications.^3,4,6^ When ferrate­(VI) technology is viewed against technology readiness levels (TRLs)—a framework for assessing technology maturity (Figure 1),^7^ research activities, funding, and resource allocation, however, remain disproportionately concentrated in the invention phase with low technology maturity (TRL 1–4). In contrast, efforts decline sharply as maturity advances into the Valley of Death (VoD) (TRL 4–7), with limited progress toward full-scale validation (TRL 7–9).

**Figure 1:**
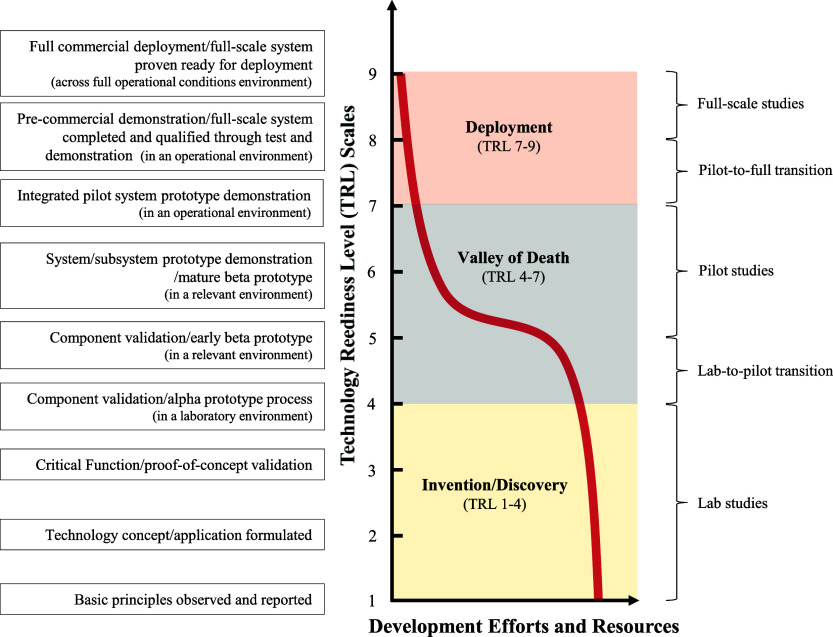
Allocation of development efforts and resources across technology readiness levels (TRLs) and the three phases of ferrate­(VI) technology development for water and wastewater treatment.

Two insights emerge. First, ferrate­(VI) technology development has entered the critical VoD—a high-risk transition stage where innovations often stall between laboratory research and practical deployment, and may ultimately fail to reach full-scale implementation.^8–10^ Second, the contrast between a long research history and limited real-world implementation indicates that the central need for ferrate­(VI) technology is not merely further laboratory discovery, but also a shift toward practical application.

A deployable ferrate­(VI) technology is a system composed of at least three subsystems—generation, treatment, and solid–liquid separation. The system has two inputs (polluted water and feedstock chemicals and/or energy for ferrate­(VI) synthesis) and two outputs (treated water and ferrate­(VI) treatment residuals (FTRs)—the sludge-like byproducts generated after solid–liquid separation) (Figure 2). The maturity of this technology is jointly determined by (1) the maturity of each subsystem and (2) their integration as a whole. We recently identified major subsystem-level obstacles to ferrate­(VI) application, including synthesis, reactor design, pH adjustment, benchmarking, and residuals management.^11^ However, these barriers were not exhaustively identified, and strategies to address them were not articulated. Meanwhile, because ferrate­(VI) research remains predominantly at the laboratory scale, system-level integration during pilot-scale development has rarely been examined. Consequently, clear pathways are lacking to translate existing subsystem-level knowledge into a deployment-ready ferrate­(VI) system.

**Figure 2:**
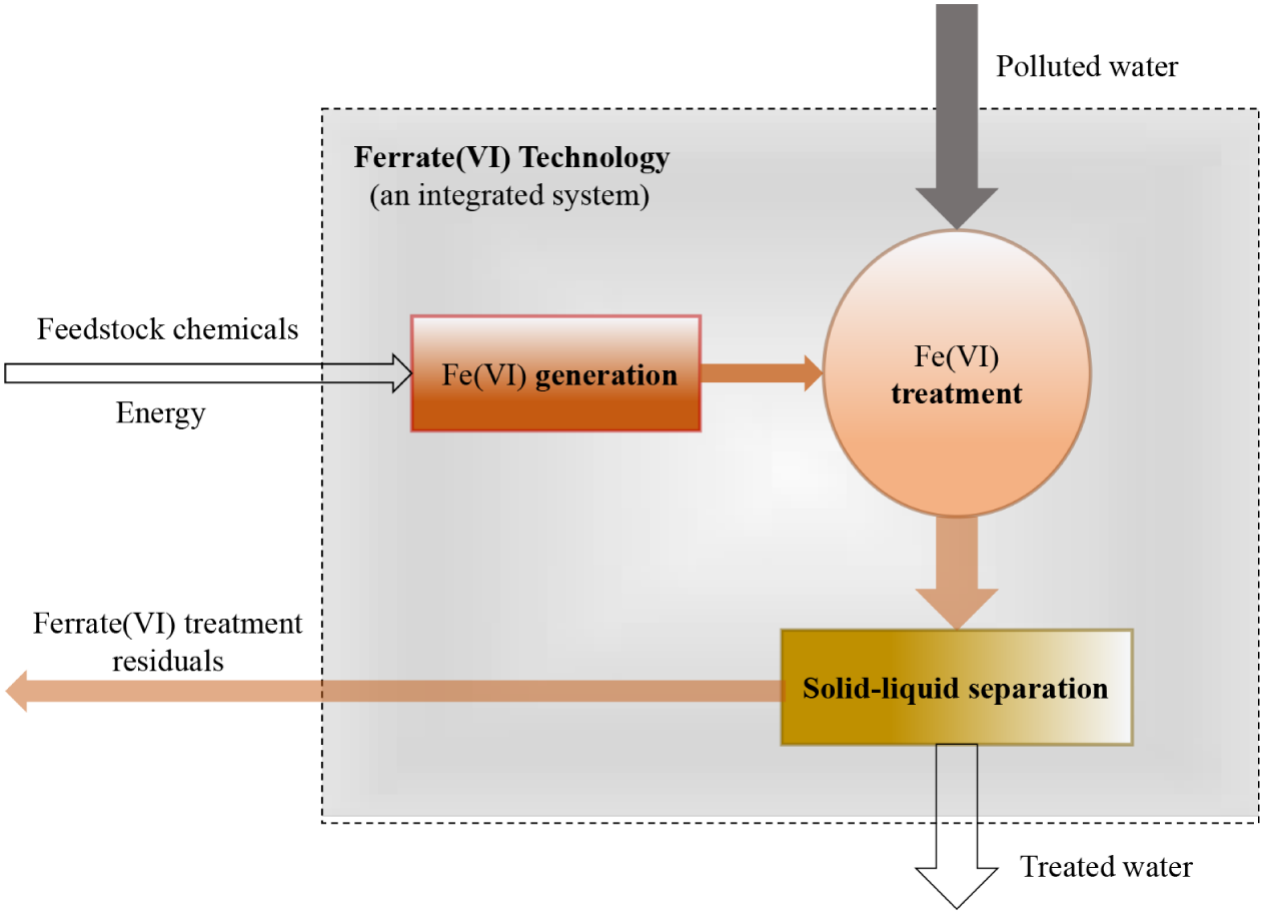
Functional architecture of a ferrate­(VI) technology system for water and wastewater treatment. The ferrate­(VI) technology is illustrated as an integrated system comprising three key subsystems: ferrate­(VI) generation, ferrate­(VI) treatment, and solid–liquid separation. The dashed lines indicate the boundaries of the ferrate­(VI) system. Within these boundaries, the generation subsystem produces ferrate­(VI), which is subsequently applied in the treatment subsystem to remove contaminants from water. The treated water then passes through a solid–liquid separation subsystem, where iron oxides formed during ferrate­(VI) reactions, likely along with associated dissolved or particulate contaminants, are removed. Although these subsystems are functionally distinct, they may not necessarily be physically isolated in practice. Across the system boundaries, there are two major inputs including polluted water and feedstock chemicals and/or energy for ferrate­(VI) synthesis. The system also produces two outputs, i.e., treated water and ferrate­(VI) treatment residuals (FTRs), which are sludge-like byproducts generated from solid–liquid separation. Although the handling of FTRs is beyond the scope of this article, their proper management is critical for the life-cycle sustainability of ferrate­(VI) technology.

To address this gap, engineering underpins the maturation of ferrate­(VI) technology with a dual role: (1) applying scientific and mathematical principles to develop practical ferrate­(VI) solutions;^12,13^ and (2) revealing challenges, not evident or observed in laboratory settings, through large-scale studies in relevant or operational environments, thereby generating feedback to refine system design or motivate new fundamental investigations.^14,15^ This perspective article presents an engineering framework to guide the development and translation of ferrate­(VI) technology in water and wastewater treatment, focusing on strategic pathways to overcome subsystem-specific barriers and challenges arising from system-level integration.

## 2 Ferrate(VI) Generation Subsystem: A Critical Technology Element

### 2.1 A Bottleneck

Figure 1 presents the collective maturity of ferrate­(VI) technology but does not differentiate the TRLs of individual subsystems. In practice, ferrate­(VI) generation has reached higher TRL levels (7–9) than ferrate­(VI) treatment, owing to the availability of commercial or large-scale ferrate­(VI) generators and their demonstrated use in applications (e.g., drinking water treatment and dredged-muck management).^16–19^ However, the generation subsystem constitutes the critical technology element (CTE) of the overall system—the fundamental module upon which operational success depends.^20^

Although a CTE typically refers to the subsystem with the lowest TRL, ferrate­(VI) generation remains the bottleneck due to a disconnect between technology readiness and market readiness. This misalignment limits the practical adoption of ferrate­(VI) generators, even when the technology is mature. Consequently, large-scale applications are scarce, constraining systematic investigations of downstream subsystems at pilot- or full-scale. As a result, ferrate­(VI) technology development continues to be trapped in the critical VoD.

To understand the misalignment, the underlying causes merit closer examination. The first cause relates to practical challenges of large-scale ferrate­(VI) generation and handling. Traditionally, to facilitate laboratory-controlled mechanistic and kinetic studies, bench-scale synthesis produces dry, stable, and high-purity potassium ferrate­(VI) (K_2_FeO_4_) powders through wet oxidation with precursor oxidants (e.g., hypochlorite (OCl^–^) and ozone (O_3_)) or electrochemical oxidation, followed by purification, solid–liquid separation, and drying steps.^21–32^ However, this procedure is labor-intensive, lengthy, and costly, involving heavy consumption of organic solvents and expensive potassium hydroxide (KOH; ∼$700–$930/ton). To address these limitations, large-scale ferrate­(VI) production has shifted toward generating concentrated sodium ferrate­(VI) (Na_2_FeO_4_) stock solutions to support sustained, high-capacity on-site ferrate­(VI) supply. Although this approach uses the same fundamental oxidation processes as laboratory synthesis, it deliberately excludes purification, filtration, and drying steps to streamline production. It generates Na_2_FeO_4_ rather than K_2_FeO_4_, leveraging the lower cost of sodium hydroxide (NaOH; ∼$360–$470/ton) and the higher solubility of Na_2_FeO_4_, which supports more concentrated stock solutions and avoids ferrate­(VI) precipitation. Nevertheless, large-scale, on-site ferrate­(VI) generation still faces three practical challenges.

First, Storage-Related Instability: On-site generation of ferrate­(VI), similar to O_3_ and chlorine dioxide (ClO_2_), mitigates losses due to chemical instability during use. However, an intermediate ferrate­(VI) storage unit is needed to buffer fluctuations in production rate and treatment demand. This storage inevitably exposes ferrate­(VI) to self-decomposition, which is accelerated by high ferrate­(VI) concentrations (tens of g/L) in stock solutions.

Stabilization of ferrate­(VI) in stock solutions has been explored and, in some cases, practiced using one or more of the following approaches, with reported stability extending from hours to more than 10 days;^16,33–35^ however, their effectiveness should be evaluated with associated trade-offs and potential drawbacks. The most common approaches include the use of high alkali concentrations and low storage temperature. Maintaining a high alkali concentration in stock solutions slows ferrate­(VI) self-decomposition and, in the presence of additional oxidants (e.g., OCl^–^), may enable *in situ* regeneration of ferrate­(VI). However, operation at highly alkaline conditions (∼several tens of percent NaOH w/w) results in substantial alkali consumption and depends on corrosion-resistant equipment and infrastructure, thereby increasing operational costs and safety risks. Moreover, such highly alkaline solutions may elevate the pH of treated water, potentially requiring post-treatment adjustment and interfering with pH-sensitive downstream processes such as coagulation. Low-temperature storage (e.g., −6 to 5 °C) represents another effective stabilization strategy,^16,36,37^ but it needs specialized temperature-control devices (e.g., cooling units and insulated tanks) and incurs additional energy consumption. Ensuring uniform temperature within storage tanks is also difficult, increasing localized risks of accelerated ferrate­(VI) decomposition or salt (e.g., NaOH) precipitation.

In addition, chemical additives have been investigated for ferrate­(VI) stabilization. One class includes oxidants (e.g., OCl^–^, O_3_, hydrogen peroxide, persulfate, and peracetic acid).^33–35^ They, when externally introduced or present due to overdosing during synthesis, may oxidize Fe­(III) produced from ferrate­(VI) decay back to Fe­(VI). A different class includes inorganic salts such as perchlorate, carbonate, bicarbonate, orthophosphate, metaphosphate, and pyrophosphate, which can stabilize ferrate­(VI) through ionic strength effects or complexation. However, their use may introduce unintended consequences. For example, perchlorate is itself an environmental contaminant,^38,39^ while phosphate addition may increase phosphorus loading in water,^40^ potentially requiring additional removal or contributing to downstream eutrophication.

Second, Undesirable Feedstock Residuals: Another issue is the presence of undesirable feedstock residuals in stock solutions, particularly Na^+^ and chlorine species, because they are typically used in large excess relative to their stoichiometric requirements. For example, applying an electrochemically produced ferrate­(VI) stock solution (5 g/L Fe­(VI) in 14 M NaOH) at 5 mg/L Fe­(VI) would introduce approximately 322 mg/L Na^+^ into treated water, far exceeding the U.S. health advisory level of 20 mg/L for sensitive populations and the taste threshold of 30–60 mg/L.^41^ When concentrated Na_2_FeO_4_ solutions are used for agricultural water reuse, excess Na^+^ can cause soil dispersion and reduce soil permeability.^42,43^ Moreover, because wet-oxidation stock solutions may also contain residual chlorine-based oxidants (e.g., OCl^–^),^11,44^ these oxidant residuals can react with organic constituents in the water matrix, such as natural organic matter (NOM), forming harmful DBPs (e.g., trihalomethanes).^45^

Third, High Energy Demand and Low Current Efficiency: The energy-intensive nature of ferrate­(VI) synthesis represents a separate obstacle when electrochemical generation is applied. Since the oxidation of Fe­(II) or Fe­(III) to Fe­(VI) is thermodynamically unfavorable, substantial energy input is required during electrochemical synthesis (0.7–several kWh/mol Fe­(VI)),^30,31,46–50^ exceeding the energy demands of synthesizing O_3_ (<0.5 kWh/mol) and chlorine (0.20–0.25 kWh/mol).^51^ Furthermore, energy efficiency declines significantly during operation. The current efficiency typically decreases from ∼70% at the onset of electrolysis to <30% as the synthesis progresses,^25,27,47,49,52–61^ due to anode passivation and accelerated ferrate­(VI) decomposition at higher concentrations. Consequently, achieving target Fe­(VI) concentrations often extends electrolysis into periods of low energy efficiency, thereby increasing overall energy demand.

The second cause is a fundamental paradox. Substantial feedstocks, energy, labor, and time are invested to produce ferrate­(VI), a strong oxidant, rather than directly applying its precursor oxidants in wet oxidation synthesis or the oxidizing environments generated in electrochemical systems. This trade-off is justified only if ferrate­(VI) offers distinct advantages. However, the value of ferrate­(VI) relative to direct chemical or electrochemical oxidation routes remains unclear, because systematic, context-specific comparisons are lacking. Consequently, the practical justification for ferrate­(VI) synthesis cannot yet be fully established.

### 2.2 Overcoming Synthesis Challenges

Past and current research has largely prioritized ferrate­(VI) treatment over the development of scalable and market-viable synthesis technologies. Because ferrate­(VI) generation is the system’s CTE, more focus should be directed toward advancing synthesis to overcome barriers related to both technological constraints and limited industrial adoption.

#### 2.2.1 Addressing Technological Constraints

An ideal ferrate­(VI) generation subsystem ensures continuous, cost-effective, and reliable production of ferrate­(VI) with adequate stability, minimal residual impacts, and sufficient modularity to support diverse applications. This vision can be realized by refining existing synthesis methods and developing new approaches. In the current ferrate­(VI) generation methods, wet oxidation is relatively energy-efficient and operationally simple, but inflexible postconstruction and reliant on continuous chemical input. Electrochemical synthesis, on the other hand, offers modular scalability, renewable energy compatibility, and real-time control, but it is energy-intensive and requires periodic electrode maintenance. Both methods share common challenges in minimizing Na^+^ residuals while achieving cost-effective ferrate­(VI) stabilization. Reducing alkali usage can lower both costs and sodium levels, but this decision must be balanced against the consequences of reduced alkaline concentration, which may weaken the suppression of side reactions (e.g., Fe­(III) precipitation), shift oxidation equilibria toward the less stable HFeO_4_^–^, and accelerate ferrate­(VI) decomposition during storage and delivery. Alternatively, replacing NaOH with KOH can avoid sodium residuals; however, the substitution entails trade-offs, including higher reagent cost and lower maximum ferrate­(VI) concentrations achievable in stock solutions due to the lower solubility of K_2_FeO_4_. Unlike Na^+^, potassium ions (K^+^) in drinking water generally pose low health concern.^62^ Moreover, it may be agriculturally preferable during agricultural reuse because it functions as a macronutrient with fewer negative soil effects.^63^ However, excessive K^+^ may still introduce constraints, including potential health risks for individuals with kidney disease^62^ and the increase in total dissolved solids.

To estimate residual cation loading during ferrate­(VI) applications, we propose a new indicator, R—the residual cation-to-ferrate ratio—for ferrate­(VI) stock solutions. R is defined as the mass ratio of the cation derived from the base to ferrate­(VI) generated in the stock solution (eq 1).

$$R=\frac{\mathrm{residual cation mass in the stock solution}\left(\frac{\mathrm{mg}}{L}\right)}{\mathrm{Fe}(\mathrm{VI})\;\mathrm{mass in the stock solution}\left(\frac{\mathrm{mg}}{L}\mathrm{as Fe}\right)}=\alpha \frac{{[\mathrm{base}]}_{\mathrm{stock}}}{{[\mathrm{Fe}\left(\mathrm{VI}\right)]}_{\mathrm{stock}}}$$ 1

where α is the atomic weight ratio of the residual cation to iron (α = 0.41 for Na^+^ and 0.70 for K^+^); and, [base]_stock_ and [Fe­(VI)]_stock_ are the molar concentrations of the base introduced and ferrate­(VI) generated in the stock solution (mol/L), respectively. Using a similar approach, new metrics can be developed to quantify other solutes (e.g., stabilizers) relative to generated ferrate­(VI).

Practically, *R* represents the mass of residual cation introduced from the base per unit mass of ferrate­(VI) dosed as Fe. Accordingly, the mass concentration of cation introduced into treated water (*C*_cat_) can be estimated as

2 $$C_{\mathrm{cat}}=R\cdot \mathrm{Fe}\left(\mathrm{VI}\right)$$

where Fe­(VI) is the applied ferrate­(VI) dose expressed as Fe (mg/L).

Other technical barriers are method-specific. In wet oxidation, increasing yields can improve oxidant utilization and reduce residuals. Avoiding chlorine-based oxidants minimizes DBP formation, while recycling oxidant residuals enhances production efficiency and cost-effectiveness. In contrast, electrochemical synthesis encounters typical challenges common to electrochemical treatment in general, such as electrode fouling, competitive oxygen evolution, high electrolyte demand, and byproduct formation.^64^ Advancing electrode materials, cell design, and modularity is therefore a solution to support large-scale deployment.

Exploring innovative and potentially disruptive ferrate­(VI) generation strategies represents another pathway to technical advancement. *In situ* electrochemical ferrate­(VI) generation at circumneutral pH, where ferrate­(VI) is produced and used in place,^65–69^ is one such attempt. However, at circumneutral pH, ferrate­(VI) becomes highly unstable, while Fe­(II) and Fe­(III) readily precipitate, reducing iron availability, limiting mass transport to the anode, and fouling electrodes. These effects result in low synthesis yields (typically <25%) and low Fe­(VI) concentrations.^65,66^ Because ferrate­(VI) preferentially reacts with abundant matrix organics like NOM,^70–73^ this *in situ* synthesis approach also leads to low degradation efficiencies for targeted contaminants of emerging concern (CECs) at trace levels. Meanwhile, these electrochemical systems can generate other oxidants, such as hydroxyl radicals (•OH), and drive direct electrochemical oxidation. The relative contributions of ferrate­(VI) versus these alternative oxidants have not been comprehensively evaluated. If the role of ferrate­(VI) is minor, this system may function more as a general electrochemical oxidation process than a ferrate­(VI)-driven treatment.

The technological innovation in ferrate­(VI) synthesis also requires re-examination of a fundamental assumption—whether high ferrate­(VI) yields, typically achieved through energy-intensive and operationally complex processes, are necessary for practical applications. As a low-cost coagulant and potential activator, ferric (Fe­(III)) salts have been jointly used with ferrate­(VI) to accomplish comparable or even superior treatment performance relative to ferrate­(VI) alone.^74,75^ These findings imply a potentially promising strategy: generating ferrate­(VI) at modest yields while retaining Fe­(III) (the ferrate­(VI) precursor) in the stock solution may offer a more cost-effective, energy-efficient, and operationally practical pathway for real-world implementation.

#### 2.2.2 Fostering Industrial Adoption

Industrial adoption of large-scale ferrate­(VI) generation should be promoted. A key step is to determine under which conditions ferrate­(VI) outperforms its precursor oxidants or direct electrochemical oxidation. To this end, comparative assessments should be both multidimensional and application-specific. Accordingly, detailed comparisons with alternative oxidants, including those used (e.g., OCl^–^ and O_3_) and those not involved (e.g., •OH) in ferrate­(VI) production, are presented in the treatment subsystem section.

Codesign is also needed to foster the adoptability of ferrate­(VI) generators. Codesign is an iterative process in which researchers and diverse stakeholders, ranging from operators and utility engineers to regulators and industry partners, engage in collaborative actions (e.g., knowledge and information sharing, joint problem identification, iterative prototyping, shared decision-making, and continuous feedback cycles) to develop deployable ferrate­(VI) generation subsystems.^76–79^ This approach helps uncover hidden constraints, translate them into design parameters, and guide the production of ferrate­(VI) in an affordable, low-energy, and simple-to-operate manner, while addressing generation challenges noted above. For example, researchers and utility engineers could jointly prototype a ferrate­(VI) generator that integrates with existing chemical dosing and control devices, allowing operators to evaluate compatibility with plant automation and industrial operation.

## 3 Treatment Subsystem: Core of Ferrate(VI) Technology

### 3.1 Engineering Ferrate­(VI) Treatment

Treatment is foundational to ferrate­(VI)-based water systems. Five decades of laboratory research have built a scientific foundation for the application of ferrate­(VI) in water and wastewater treatment, as detailed in prior reviews.^6,80–83^ Building on this foundation, this section focuses on the engineering aspects required to advance ferrate­(VI) treatment toward practical implementation.

#### 3.1.1 Comparative Evaluation Framework for Ferrate­(VI) Treatment

A key challenge is the lack of systematic, side-by-side comparisons with alternative oxidants, which limits the ability to determine under which conditions ferrate­(VI) is advantageous. As discussed above, some of these comparable oxidants are also used for ferrate­(VI) synthesis (e.g., OCl^–^ and O_3_), whereas others, such as •OH-based advanced oxidation processes (AOPs), are not. Addressing this gap requires both comprehensive and context-specific comparisons. Here, “comprehensive” refers to evaluation across multiple dimensions, such as technical performance, energy demand, cost, operational complexity, and residuals management, reflecting distinct technical and nontechnical characteristics of these oxidation processes. For example, ferrate­(VI) is a selective oxidant that preferentially degrades electron-rich organic compounds, with a standard reduction potential of up to 2.2 V comparable to ozone (2.07 V),^6^ but less reactive than unselective •OH. It is inherently safer with the production of fewer DBPs.^84–86^ Beyond oxidation, its multifunctionality enables removal of certain dissolved contaminants (e.g., orthophosphate) and particulates^87^ via precipitation, adsorption, and coagulation, which O_3_, •OH, or many other oxidants do not typically achieve.^88^ However, the resulting iron (hydr)­oxides require separation, while the final FTRs need special management. Moreover, unlike OCl^–^, ferrate­(VI) cannot provide secondary disinfection during water storage and distribution due to the lack of a lasting disinfectant residual.

Beyond multidimensional comparisons, comparative assessments of ferrate­(VI) and its potential competitors should be conducted under application-dependent conditions, such as water matrix composition, target contaminants, treatment goals, and system configuration. These assessments can ultimately inform context-specific decisions. For example, although ferrate­(VI) oxidation, like ozonation and AOPs, effectively removes diverse CECs in secondary effluent, it may be more competitive when effluent phosphate is limited, as required in many U.S. states.

Based on these assessments, an evaluation framework can be established to develop engineering decisions, reduce uncertainty, and enable evidence-based comparisons. This framework assists stakeholders by serving as a decision-support and screening tool for consulting engineers and utilities in selecting ferrate­(VI) applications, informing regulators of the benefits and risks, and identifying application niches for technology developers.

#### 3.1.2 Reactor Design and the Laboratory-to-Real-World Discrepancy

While substantial research has focused on ferrate­(VI) treatment, most studies remain at the laboratory scale (TRL 1–4), with limited exploration at pilot scale. One underlying reason is the aforementioned challenges in ferrate­(VI) generation. Consequently, critical engineering aspects of the treatment subsystem, such as reactor design, process scale-up, energy demand, and economic feasibility, are largely unexplored, constraining progress toward pilot-scale implementation and overall system advancement.

To address this issue, two key dimensions are particularly important: reactor design and the gap between laboratory and real-world operating conditions. Reactor design is inherently inseparable from higher system-level configuration, as discussed later in Section 5. The other challenge arises from the discrepancy between controlled laboratory conditions and complex real-world environments. Variations in solution chemistry (e.g., pH) and operational conditions (e.g., temperature) can alter treatment outcomes because ferrate­(VI) behavior is highly environment-dependent. Special attention should be given to pH and acidity (i.e., the capacity to react with a strong base to a designated pH).^89^ While the effects of pH on ferrate­(VI) chemistry are well-known—higher pH increases Fe­(VI) longevity and lowers reactivity—the role of acidity in practical ferrate­(VI) treatment has been neglected.^90^ Unlike highly buffered laboratory systems, real waters typically exhibit dynamic pH evolution after ferrate­(VI) dosing, reflecting the interplay between the release of OH^–^ from ferrate­(VI) protonation and self-decay and the buffering of acidity-contributing species such as carbonate, NOM, and ammonium.^11,90^ As a result, the temporal, system-specific pH profile is jointly governed by initial pH and acidity, highlighting their roles in translating ferrate­(VI) technology to full-scale applications.

### 3.2 Ferrate­(VI) Activation: A Silver Bullet?

Ferrate­(VI) activation enhances oxidation by generating and/or stabilizing more reactive intermediates^91–93^ through energy input (e.g., UV irradiation)^94,95^ or the addition of activators (e.g., sulfite (SO_3_^2–^),^74,82,91–105^ thiosulfate (S_2_O_3_^2–^),^106^ metal ions,^107^ and biochar^102,103^ ). Since its discovery, this approach has rapidly become a research hotspot, as it provides a simple means to boost ferrate­(VI) reactivity and accelerate degradation of persistent CECs from hours to minutes or seconds.^96,98,108^ However, the occurrence of ferrate­(VI) deactivation and the potential impairment of disinfection under activation conditions highlight the need for engineering considerations in translating activation strategies into practice.

Importantly, “activation” represents only one regime of broader ferrate­(VI) modulation, as deactivation may coexist. Ferrate­(VI) reactivity in the presence of an activator depends on reaction conditions, particularly [activator]:[Fe­(VI)]. Within certain ranges, activation may vary in extent or even shift to a “deactivation” state, where CEC degradation is lower than with ferrate­(VI) alone.^109,110^ This behavior arises from complex interactions, such as enhanced scavenging of ferrate­(VI) when a reductive activator (e.g., SO_3_^2–^) is in excess. Thus, so-called activators are more appropriately viewed as reactivity modulators (RMs). Accordingly, the conventional ferrate­(VI)-activator system is reframed broadly as a ferrate­(VI)-RM system that can shift between activation and deactivation states depending on reaction conditions.^111^

In practice, steering the ferrate­(VI)–RM system toward activation is critical . Achieving this goal requires understanding the RM mode, whether engineered or passive. Ferrate­(VI) reactivity can be modulated not only by externally added chemical agents (engineered modulation), but also by solutes inherently present in water matrices (e.g., ammonium and bicarbonate) (passive modulation).^82,104^ Among engineered modulators, for example, SO_3_^2–^ is promising due to its wide industrial use for dechlorination and its demonstrated effectiveness in ferrate­(VI) activation. In this context, a key strategy for engineered activation is to control initial [activator]:[Fe­(VI)], as deactivation may occur when the ratio falls outside an optimal range. In contrast, passive modulation takes place unintentionally. When deactivation happens, mitigation is required, such as pretreatment to remove interfering solutes (e.g., pH adjustment and air sparging to reduce high ammonium concentrations during ferrate­(VI) degradation of pharmaceuticals in urine).

However, ensuring consistent activation becomes difficult when multiple CECs are present, as each may require distinct optimal activation conditions (e.g., [RM]:[Fe­(VI)]). Under a given condition, only a subset of CECs may fall within the optimal activation regime, while others remain suboptimal or even in the deactivation state. To manage this complexity, engineering strategies may prioritize high-priority contaminants or implement staged ferrate­(VI) activation steps, each tailored to specific groups of CECs.

Besides the reactivity modulation, another engineering consideration is the trade-off between CEC degradation and disinfection. Activated ferrate­(VI), though enhancing CEC degradation, may compromise disinfection because the formation of more reactive but short-lived oxidants reduces the effective oxidant exposure (∫[Oxidant] d*t*).^112^ Therefore, activation should be applied with caution when waterborne pathogens, rather than CECs, are the primary contaminants. When both contaminant groups are of concern, treatment conditions should be optimized to identify an operational window that balances both objectives. Alternatively, a staged design can provide a solution in which ferrate­(VI) alone and activated ferrate­(VI) are applied in separate stages for disinfection and CEC removal, respectively.

Additionally, a critical question is the extent to which practical ferrate­(VI) activation should be pursued. Activated ferrate­(VI) can generate high-valence iron species (e.g., Fe­(V) and Fe­(IV)) and free radicals (•OH and SO_4_^•–^).^82,96,97,104,113–115^ The dominant oxidant species depend on activation methods and reaction conditions (e.g., pH).^116^ In some cases, activation strategies, such as the addition of peroxymonosulfate (PMS), intentionally promote the formation of •OH and •SO_4_^2–^, effectively shifting ferrate­(VI) treatment toward free radical-driven AOPs.^100^ However, such transformations require scrutiny, as other AOPs (e.g., UV/H_2_O_2_) can often be implemented more directly and cost-effectively.^117^ Moreover, promoting free radicals or Fe­(V)/Fe­(IV) improves reactivity but reduces oxidative selectivity, increasing inefficient oxidant consumption in matrices rich in electron-rich organic matter. Therefore, the added value of ferrate­(VI) activation should be evaluated against conventional AOPs in a comprehensive, context-specific manner.

## 4 Solid–Liquid Separation: An Overlooked Subsystem

Ferrate­(VI) treatment produces iron (hydr)­oxide particles that can further interact with solutes (e.g., arsenic)^87,118–120^ and other particulates,^121–123^ enabling additional contaminant removal. A solid–liquid separation step is ultimately required to remove the resulting solids. This subsystem is indispensable for removing dosed iron, newly formed iron (hydr)­oxides, and captured contaminants, thereby ensuring that treated water meets its intended quality goals. Otherwise, turbidity may be problematic, such as violating drinking water standards or clogging sprinklers during wastewater reclamation for irrigation, while residual iron (hydr)­oxides may cause aesthetic issues, such as reddish discoloration.

Notably, although the removal of ferrate­(VI)-derived solids has occasionally been investigated in laboratory- or pilot-scale studies, this step is unlikely to limit technology readiness, because solid–liquid separation is widely applied in the water industry. These resulting solids, whether readily settleable or not, span a size range from the nanometer to millimeter scale. Such particles can be effectively removed using mature technologies, such as sedimentation, rapid sand filtration, and membrane filtration. However, the current challenges for this subsystem lie in the limited knowledge of the composition and characteristics of these solids, which in turn influence the choice of solid–liquid separation strategies in different application scenarios.

One knowledge gap pertains to the composition and structure of these solids. Prior studies have reported inconsistent or even conflicting observations on the mineralogical identity and crystallinity of ferrate­(VI)-derived iron (hydr)­oxides, ranging from amorphous structures^124,125^ to various crystalline forms (e.g., hematite (α-Fe_2_O_3_), maghemite (γ-Fe_2_O_3_), and lepidocrocite (γ-FeOOH)),^87,120,126^ or mixtures of both.^11,121,127^ Such divergences reflect the mechanistic complexity and dependence on water quality conditions. Particularly, most laboratory studies evaluate ferrate­(VI) treatment solids in particle-free water^87,126,128,129^ or after original particles are filtered.^70^ While this setting facilitates mechanistic probing, it raises the question of whether the solids formed are representative of those produced in real-world waters.

This concern arises for three reasons. First, the absence of original particles can obscure pathways that influence particle formation and characteristics, such as surface-mediated nucleation and growth.^130,131^ Second, in particle-rich matrices (e.g., municipal wastewater), ferrate­(VI)-derived iron (hydr)­oxides become incorporated into complex and often more abundant preexisting particulates, as observed in ferrate­(VI)-enabled chemically enhanced primary treatment.^132^ Third, when ferrate­(VI) treatment is applied as an auxiliary step, it may share units with conventional processes that generate solids (e.g., coagulation flocs), leading to mixed solids that are more complex than those formed in particle-free water. Consequently, insights from studies conducted in particle-free water provide only limited information about the overall solids formed in such complex matrices. This compositional and structural complexity challenges accurate solid characterization.

Another gap is the limited understanding of ferrate­(VI)-enabled coagulation. A long-standing assumption is that ferrate­(VI) produces Fe­(III) that simply mimics conventional coagulation by externally dosed Fe­(III). However, this is untenable. Laboratory studies reveal different coagulation behaviors—some report effective floc formation,^121,133–135^ whereas others observe elevated turbidity and nanoparticle generation that hinder downstream separation.^122,126,135^ Poor aggregation is often attributed to increased negative surface charge or colloid restabilization,^122,135^ but the role of Fe­(III) species formed *in situ* from ferrate­(VI) decay has been largely overlooked. Unlike externally dosed Fe­(III), which hydrolyzes to positively charged mono- and polynuclear species to drive charge neutralization,^136^ ferrate­(VI) decays via anionic iron-oxo intermediates (Fe­(VI), Fe­(V), and Fe­(IV)). Fe­(III) produced may initially remain in negatively charged coordination complexes, inhibiting the development of cationic hydrolysis species and thereby limiting charge neutralization. Additionally, changes in operational conditions (e.g., activation with sulfite) may impair particle aggregation and settling.^137^ These uncertainties translate into variability in two key particle properties governing solid–liquid separation: settleability and particle size distribution. Consequently, selecting effective downstream separation processes becomes more challenging.

When coagulation performs effectively, ferrate­(VI) can generate dense, settleable flocs that can be effectively removed through gravity-driven separation processes (e.g., sedimentation), followed by depth filtration (e.g., rapid sand filters). However, when coagulation fails, ferrate­(VI) may produce fine, nonsettleable particles.^122^ Removal of these small suspended particles typically relies on more effective separation processes such as size-exclusion methods (e.g., microfiltration). However, in some cases—for example, at low ferrate­(VI) doses—(hydr)iron oxide particles may be sufficiently small to pass through microfiltration membranes. Such unforeseen increases in particle loading introduce additional challenges. More stringent, but more expensive and energy-intensive, separation options (e.g., ultrafiltration) may be required, or the particles may need to be treated as additional particulate contaminants and removed through supplemental coagulant dosing. Additionally, innovative separation strategies have been explored. For example, magnetic separation has been proposed for removing ferrate­(VI)-induced magnetic iron oxides such as maghemite (γ-Fe_2_O_3_).^87,120^ However, caution is warranted due to its scalability constraints, high costs, and the coexistence of nonmagnetic iron phases and diverse background particulates.^121,126,138,139^

## 5 The Next Frontier: System-Level Integration

Current research predominantly advances ferrate­(VI) subsystems independently, leaving system-level integration relatively underexplored. As ferrate­(VI) technology progresses beyond subsystem development, system-level integration challenges will inevitably arise. Here, we highlight key challenges anticipated for this transition.

These challenges fall into two system-level implementation configurations, each involving different treatment reactor designs at the subsystem level. The first is the auxiliary ferrate­(VI) configuration (Figure 3a), in which ferrate­(VI) is incorporated into existing water or wastewater treatment trains as a pre-,^132^ intermediate-,^140^ or polishing step,^141^ with minimal infrastructure modification (e.g., requiring corrosion-resistant materials where needed). In this configuration, the ferrate­(VI) system often shares treatment and/or solid–liquid separation units with conventional processes,^17,37,134^ leveraging existing infrastructure (e.g., rapid mixers for preoxidation and coagulation)^134,142^ to reduce capital costs while producing mixed residuals, including FTRs, for disposal.^134^ However, this plug-in approach primarily exploits only one function of ferrate­(VI), particularly chemical oxidation, to assist existing processes such as removing CECs. Consequently, although this configuration lowers implementation barriers, it underutilizes ferrate­(VI)’s multifunctionality.

**Figure 3:**
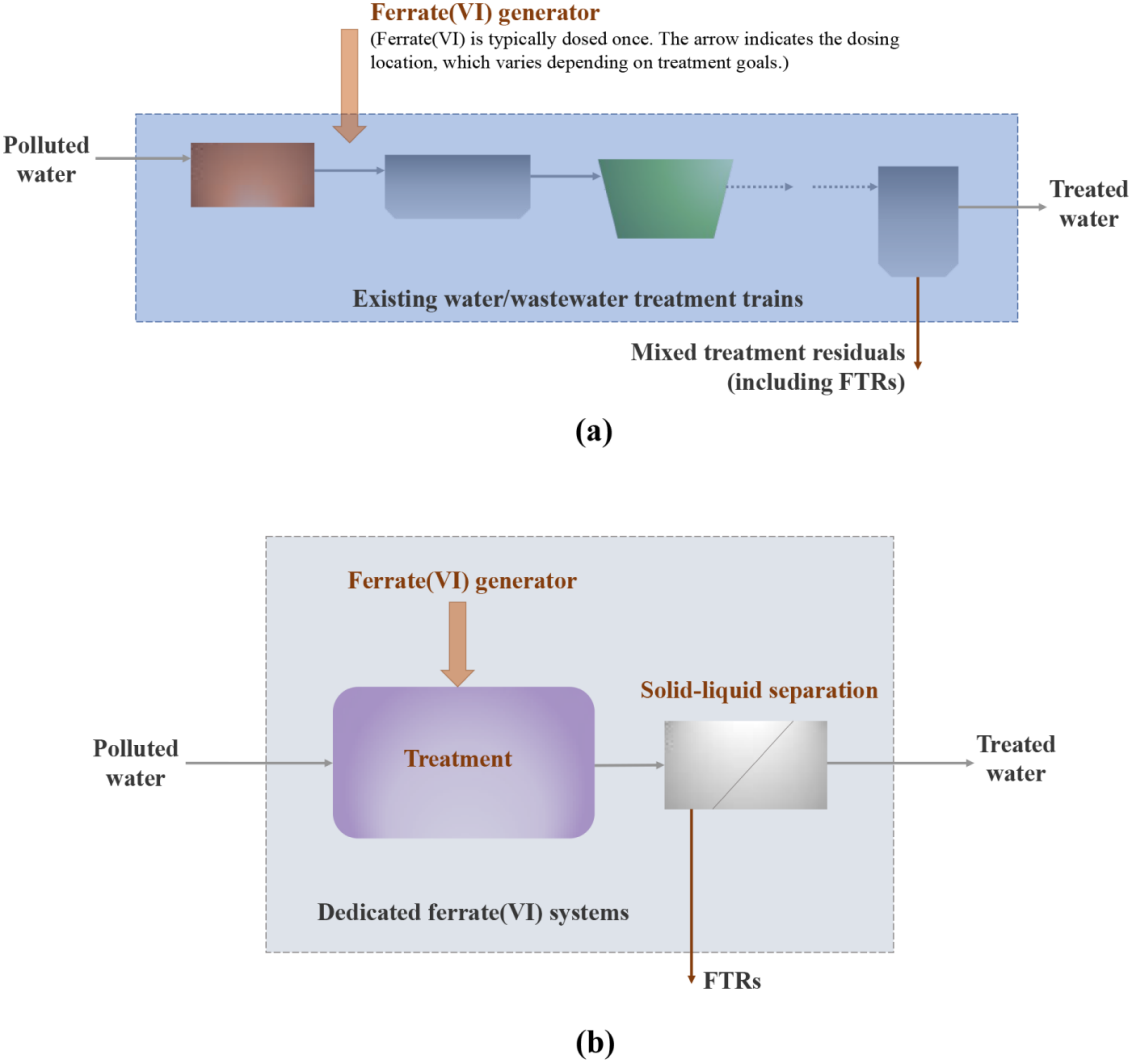
Two system-level ferrate­(VI) implementation configurations: (a) Auxiliary ferrate­(VI) configuration: ferrate­(VI) is introduced as a pre-, intermediate-, or post-treatment into existing water or wastewater treatment trains, where it shares infrastructure with conventional processes and produces mixed treatment residuals (including FTRs); (b) Standalone ferrate­(VI) configuration: ferrate­(VI) is applied within an independent treatment system comprising ferrate­(VI) dosing, treatment, and solid–liquid separation units, generating separate FTR streams.

Under this auxiliary configuration, a challenge is the misalignment between ferrate­(VI)’s optimal operating conditions and the hydraulic, operational, and infrastructure constraints of existing treatments processes. Because a ferrate­(VI) system, as a supporting role, is embedded within predesigned systems, its operation is often influenced by conditions optimized for conventional processes rather than ferrate­(VI) treatment itself. This constraint limits ferrate­(VI) performance and introduces trade-offs between process compatibility and treatment efficiency. Addressing this challenge requires case-specific engineering solutions that balance ferrate­(VI) performance and system constraints. For example, in ferrate­(VI) preoxidation for drinking water treatment, ferrate­(VI) dosing can increase downstream pH beyond the optimal range for subsequent coagulation (e.g., typically 5.5–7.7 for Al­(III) and 5.0–8.5 for Fe­(III)).^136^ Consequently, pH adjustment prior to coagulant addition is required, introducing additional operational complexity and cost.

Alternatively, a standalone ferrate­(VI) configuration can be adopted (Figure 3b), in which a dedicated ferrate­(VI) system comprising three subsystems operates independently.^65–69,135,141^ In this context, the ferrate­(VI) system does not share infrastructure with existing processes and produces separate FTRs rather than mixed plant residuals. Subsystems within this configuration may be physically separated or integrated. For example, multifunctional ferrate­(VI) reactors have been proposed to combine treatment with solid–liquid separation,^143^ while *in situ* electrochemical systems integrate ferrate­(VI) generation and treatment within a single unit.^65–69^ Although this configuration requires higher capital investment due to dedicated infrastructure, it harnesses ferrate­(VI)’s multifunctionality with greater operational flexibility.

Within this configuration, single-unit ferrate­(VI) systems that integrate multiple treatment functions and solid–liquid separation are particularly attractive, as they can reduce unit operations, footprint, and operational complexity, while potentially lowering energy consumption, making them particularly suitable for small water and wastewater systems.^74,135,141,144^ However, such designs are inherently more complex due to integration challenges arising from competing requirements across treatment functions and interactions among subsystems. For example, conditions optimized for oxidative degradation of CECs may produce solids with poor settleability or unfavorable particle sizes for membrane separation. These conflicting requirements highlight that system-level integration requires coordinated design rather than simply coupling individually optimized processes, with consideration of water quality compatibility, hydraulic constraints, operational conditions, energy use, and cost, as well as nontechnical factors such as scalability and operational simplicity. To address these challenges, advanced engineering strategies are needed, such as process intensification to enable multifunctionality within one reactor and multi-objective optimization to balance trade-offs among chemical dosing, reaction kinetics, and effluent quality.^145–147^

Regardless of the configuration, FTRs, alone or combined with other treatment residuals, require appropriate management. However, research on their systematic characterization, treatment, reuse, or disposal is limited. Although outside the scope of this article, effective management of FTRs represents an important consideration for life-cycle assessment and the long-term sustainability of ferrate­(VI) technology.

## 6 Outlooks

The preceding sections highlight key obstacles and corresponding engineering strategies for ferrate­(VI) technology at both subsystem and system levels, revealing that the central challenge is not the lack of ferrate­(VI) reactivity, but the absence of integrated, deployable technology. Addressing this gap requires stronger alignment between scientific advances and engineering implementation.

Building on these insights, advancing ferrate­(VI) technology depends on progress in three dimensions. First, *maturity stage–aligned technology development*. In conventional trajectories, key contextual constraints, such as energy demand, cost, operational complexity, and residuals management, become increasingly dominant at higher TRL levels. Given that ferrate­(VI) technology is currently positioned within the VoD, a critical transition phase for technology survival, near-term efforts should prioritize derisking subsystem- and system-level uncertainties to advance through this stage, before moving toward long-term goals of full-scale deployment.

Second, *subsystem-to-system integrated design*. System-oriented integration does not simply assemble individually optimized subsystems; instead, it explicitly accounts for their interactions and resolves functional trade-offs. More importantly, system-level studies enable prototype development as entry points for pilot-scale validation, while providing critical feedback to refine ferrate­(VI) performance and reveal new mechanistic questions. System integration is therefore a key pathway through which ferrate­(VI) technology advances across the VoD.

Third, *application-specific decision guidance*. This dimension focuses on application-level decision-making for ferrate­(VI) technology deployment. Comparative evaluation against existing technologies should be conducted in a quantitative and comprehensive manner, considering both technical and nontechnical factors, to identify application niches where ferrate­(VI) provides clear advantages under different implementation configurations. Meanwhile, as technology readiness progresses, market readiness should evolve accordingly to avoid developing technologies that are technically sound but practically unacceptable, as illustrated by the current status of many large-scale ferrate­(VI) generators. From an engineering perspective, bridging this gap requires both scientific and engineering advances to improve key technology attributes, such as reducing costs and enhancing operational simplicity, as well as codesign with water managers, operators, and other stakeholders to align ferrate­(VI) technology with real-world needs and constraints.

Looking ahead, sustained engineering efforts will play a critical role in advancing ferrate­(VI) technology toward practical and scalable implementation in water and wastewater treatment. Further exploration will expand the applicability of ferrate­(VI) to address diverse water contaminants.
